# Expressed sequences tags of the anther smut fungus, *Microbotryum violaceum*, identify mating and pathogenicity genes

**DOI:** 10.1186/1471-2164-8-272

**Published:** 2007-08-10

**Authors:** Roxana Yockteng, Sylvain Marthey, Hélène Chiapello, Annie Gendrault, Michael E Hood, François Rodolphe, Benjamin Devier, Patrick Wincker, Carole Dossat, Tatiana Giraud

**Affiliations:** 1 UMR 8079 CNRS-UPS, Ecologie, Systématique et Evolution, Bâtiment 360, Université Paris-Sud, F-91405 Orsay Cedex, France; 2 UMR 5202, CNRS-MNHN, Origine, Structure et Evolution de la Biodiversité, Département Systématique et Evolution, 16 rue Buffon CP 39, 75005, Paris, France; 3 INRA, Unité Mathématique, Informatique et Génome, Domaine Vilvert, Jouy-en-Josas, F-78352, France; 4 Department of Biology, Amherst College, Amherst, MA 01002, USA; 5 Génoscope, UMR CNRS 8030, 2 Gaston Crémieux, CP 5706, 91507 Evry, France

## Abstract

**Background:**

The basidiomycete fungus *Microbotryum violaceum *is responsible for the anther-smut disease in many plants of the Caryophyllaceae family and is a model in genetics and evolutionary biology. Infection is initiated by dikaryotic hyphae produced after the conjugation of two haploid sporidia of opposite mating type. This study describes *M. violaceum *ESTs corresponding to nuclear genes expressed during conjugation and early hyphal production.

**Results:**

A normalized cDNA library generated 24,128 sequences, which were assembled into 7,765 unique genes; 25.2% of them displayed significant similarity to annotated proteins from other organisms, 74.3% a weak similarity to the same set of known proteins, and 0.5% were orphans. We identified putative pheromone receptors and genes that in other fungi are involved in the mating process. We also identified many sequences similar to genes known to be involved in pathogenicity in other fungi. The *M. violaceum *EST database, MICROBASE, is available on the Web and provides access to the sequences, assembled contigs, annotations and programs to compare similarities against MICROBASE.

**Conclusion:**

This study provides a basis for cloning the mating type locus, for further investigation of pathogenicity genes in the anther smut fungi, and for comparative genomics.

## Background

Deciphering the molecular mechanisms involved in infection is important for the control of devastating crop diseases. Furthermore, the comparison of pathogenicity-related genes from different fungi provides insight into the evolution of host-pathogen interactions, thereby advancing our understanding of host specificity, virulence, and the emergence of new diseases. Modern sequencing technologies have led to a remarkable increase in genomic data available for identifying genes by similarity searches [1]. Key genes involved in pathogenicity in several fungi have been compiled into the PHI database [2].

In the smut fungi of monocot hosts (e.g. *Ustilago maydis *and *U. hordei*, major pathogens of corn and barley, respectively), the sexual phase and the genes linked to the mating-type loci play a key role in development and pathogenicity [3]. Mating-type loci determine sexual compatibility: only individuals differing at these loci can mate. In *U. maydis*, cell recognition and fusion is regulated by a pheromone/receptor system that resides at the *a *locus. After fusion, the dikaryon is maintained and cells switch to filamentous growth if they are heterozygous for the second mating type locus, the *b *locus [4,5]. The *b *locus encodes two homeodomain proteins that function as transcriptional regulators after dimerization. The majority of sexual basidiomycete fungi possess such a system called "tetrapolar", where *a *and *b *unlinked loci (respectively called B and A in some species) are both involved in sexual compatibility and are often multiallelic [5,6]. Other members of this phylum are "bipolar", due to the *a *and *b *loci being tightly linked (e.g. in *U. hordei*, [7]) or due to one of the two mating type loci having lost their role in mating type specificity (e.g. in *Coprinellus disseminatus*, [8]). Tetrapolarity is likely ancestral [9] and promotes outcrossing as it increases the number of available mating type. The study of mating-type loci is important for understanding the infection process and the evolution of mating systems in basidiomycetes.

A widely recognized model to study host-pathogen coevolution and fungal genetics is the anther smut fungus *Microbotryum violaceum *(Pers.) Deml and Oberw. (formerly *Ustilago violacea *(Pers.) Fuckel), which is a basidiomycete, obligate parasite of more than 100 perennial species of Caryophyllaceae [10]. In plants infected by *M. violaceum*, fungal teliospores are produced in anthers and diseased plants are usually completely sterilized, the pollen being replaced by fungal spores and the stigmas and ovaries being reduced. New infections occur when fungal spores are transported from a diseased to a healthy plant by the insects that usually serve as pollinators. Once deposited on a host plant, diploid teliospores undergo meiosis and give rise to four haploid cells, two of mating type A1 and two of mating-type A2, *M. violaceum *having a bipolar mating system. Each of these post-meiotic cells can buds off yeast-like sporidia on the plant surface. New infectious dikaryons are produced only after conjugation of two cells of opposite mating-types [11]. The fungus then grows endophytically and causes perennial systemic infections.

Although *M. violaceum *is related to major crop pathogens like *U. maydis *and *Puccinia *spp., it has no impact on human activities, making it valuable for the study of natural host-pathogen coevolution, and avoiding the risk of dispersion in human crops. However, one present limitation of this model is that little genomic sequence data are available, except studies on transposable elements and on the genomic defense mechanism against the accumulation of mobile elements [12]. In particular, the mating-type locus was reticent to several cloning attempts (T. Giraud and M.E. Hood, unpublished; J. Kronstad, pers. com.) and there exist few sequences of expressed genes from *M. violaceum *in public databases. Only a few *Microbotryum *genes that contribute to hyphal development and subsequent infectious capability have been described [13].

The generation of Expressed Sequence Tags (EST) is an efficient tool to discover novel genes and investigate their expression at different developmental stages (e.g., [14,15]). Therefore, a cDNA library has been built from pools of mating haploid cells and growing infectious hyphae for a single dikaryotic isolate of *M. violaceum *collected from the host plant species *Silene latifolia*. Genes involved in mating and during early pathogenic development were expected to be expressed under these conditions because they represent the mating and infectious stages. We generated 24,128 ESTs from this library, on which we performed similarity searches in order to identify genes with functions known as important for these developmental stages.

## Results and discussion

### EST sequence analysis

The cDNA library created from poly(A)+mRNA from seven days-old mixed A1 and A2 cultures produced enough material to sequence 40,000 clones. A total of 28,430 sequences were obtained (success rate of 71%) with an average read length of 815 bp, which is similar to the EST library of *U. maydis *[14]. Some ribosomal (n = 109), mitochondrial (n = 16) and vector (n = 14) sequences were identified. After discarding them, a total of 24,128 ESTs were obtained (85% of the initial sequences). After trimming vector and low quality sequences, the average cDNA read was not very long, with 345 ± 167 bases (mean ± SD). We indeed did not select the sizes of mRNA, as recommended for normalized libraries.

**Figure 1:**
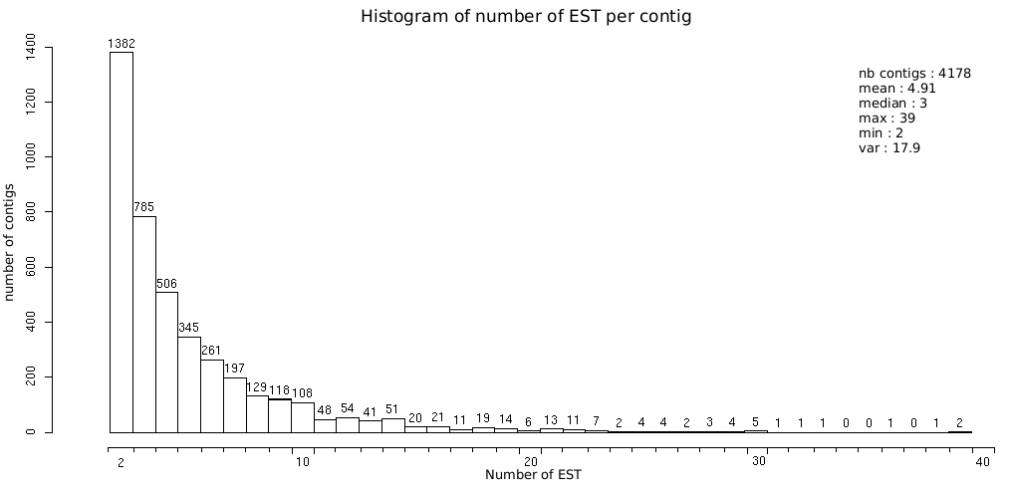
**Distribution of *Microbotryum violaceum *EST**. EST redundancy among the 7,765 unisequences obtained from a cDNA library of the basidiomycete fungus *Microbotryum vi.olaceum*. The number of ESTs is indicated above each bar.

These 24,128 ESTs were assembled into 4,178 contigs while 3,587 remained as singlets (Figure 1). This corresponds to a redundancy of 85% (number of ESTs assembled in clusters/total number of ESTs), which is very high compared to the redundancy obtained in other fungal EST libraries such as *Phytophtora parasitica *(49.5%, [16]), *Botrytis cinerea *(67%, [15]) or *Ustilago maydis *(72.3%, [14]). This does not result from a low efficiency of our normalization, but from the large scale of the present study compared to the ones cited above. Our library indeed had a size of 6.65× compared to *P. parasitica*, 3.74× compared to *B. cinerea *and 8.4× compared to *U. maydis*. The number of unisequences (i.e. all contigs and singlets) identified in our library represents 7,765 putative unique genes, which lies within the total gene number in fungi (range from 5,570 to 16,597 [17]).

### Online database: MICROBASE

A website is available with open access to the EST sequences, unisequences and annotations [18]. Several tools are made available, allowing visualising contig assemblies and performing searches on our ESTs or unisequences, by BLAST, by annotation, by function, and by sequence ID. The database was named MICROBASE, after *Microbotryum *EST database.

### Functional classification

Similarity searches performed on the 7,765 unisequences indicated that 1,953 (25.2%) had a highly significant similarity to UniProt or Genbank entries (E-value ≤ 10^-10^). Among these, 125 unisequences were similar to strictly "hypothetical" or "unknown" proteins. A total of 818 sequences (10.5%) could be classified in a putative cellular function according to the characterization scheme outlined by the Gene Ontology Consortium. In addition, 5,772 (74.3%) unisequences showed moderate to very low similarity (10^-10 ^< E-value < 10^-1^) to the UniProt and Genbank databases. A total of 125 unisequences (0.5%) had no similarity to any existing UniProt nor Genbank entry ("orphans"). This high frequency of genes without significant BLAST hit is similar to previous fungal EST libraries (e.g., [14,15]). In some cases, this lack of similarity to protein database entries could be due to the sequence being derived from the 5' or 3' untranslated region of the cDNA [19]. Among the 1,953 sequences that had a highly significant similarity to Genbank entries, 93.48% had their most significant hit against fungal sequences and 4.79% against sequences from other organisms: animals (1.6%), plants (2.58%), protozoa (0.37%) and bacteria (0.24%).

**Figure 2:**
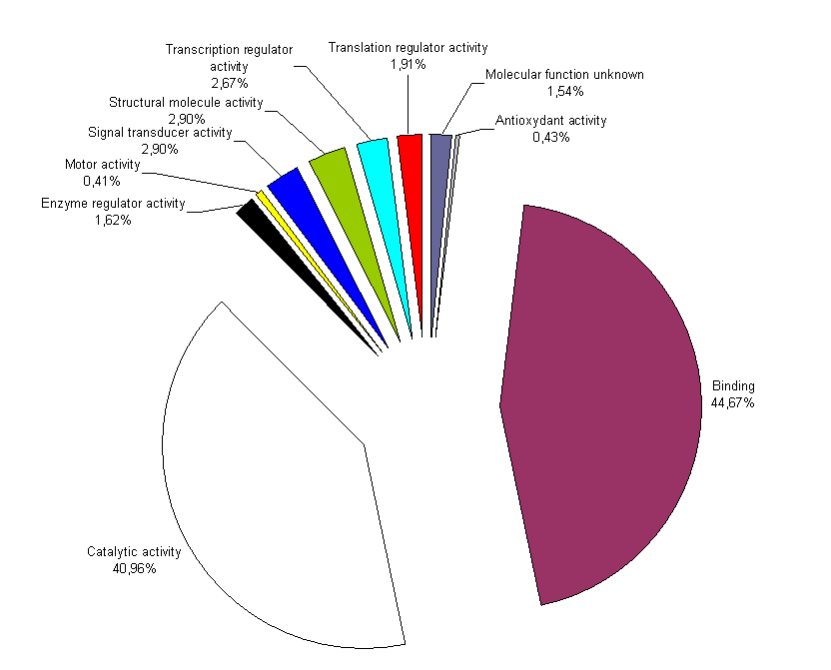
**Molecular function categories of *Microbotryum violaceum *sequences**. Distribution of the 797 contigs and singlets having a significant blast hit in public databases into molecular function class according to the Gene Ontology classification.

Regarding the Gene Ontology classes in our *M. violaceum *EST library, the molecular function class was the most abundant (37.65%), followed by the cellular component class (30.68%), and by the biological process class (16.35%). Whitin the molecular function class, sequences classified in catalytic and binding activities functions were the most abundant (Figure 2). We found also 33 unisequences with significant similarity to genes belonging to, or linked to, the mating-type loci of other basidiomycetes (see the Table in Manual Annotations in the "Annotations" section at MICROBASE) and 70 unisequences with significant similarity to genes that have been shown experimentally to be involved in pathogenicity in other fungi according to the PHI-base [2] (Table 1, see also Manual Annotations in the "Annotations" section at MICROBASE). In addition, 148 unisequences (15.31%) showed significant similarity to transposable elements.

**Table 1:** Contigs of *Microbotryum violaceum *blasting significantly to the pathogenicity-related genes reported in the PHI-database

**Putative function**	**Contig or singlet ID**	**E-value**	**EMBL accession**	**PHI accession**	**Gene Ontology category**	**Gene Ontology class**
ABC-transporter	1816	6,00E-53	BAC67162	391	Transporter activity/catalytic activity	Molecular function
	211	1,00E-16	AAK15314	310	Transporter activity/catalytic activity	Molecular function
Acetolactate synthase	3362	8,00E-20	AAR29084	358	Catalytic activity	Molecular function
Adenylate cyclase	pr0aaa104yj02scm1.1	4E-08	AAG60619	241	Catalytic activity	Molecular function
ATP molecular dependent chaperone	342	1,00E-60	AAA02743	463	Binding	Molecular function
Benomyl/methotrexate resistance	547	2E-11	CAA37820	26	Transporter activity	Molecular function
capsule protein	pr0aaa63yn21scm1.1	8,00E-15	BAC76819	139	Transporter activity	Molecular function
Carnitine acetyl transferase	1367	2E-14	AAB88887	120	Catalytic activity	Molecular function
	830	1E-12	AAB88887	120	Catalytic activity	Molecular function
Chitin synthase	1149	1,00E-31	AAC34496	236	Catalytic activity	Molecular function
	pr0aaa19yo09scm1.1	1E-11	AAC35278	237	Catalytic activity	Molecular function
	2395	9,00E-27	AAT77184	337	Catalytic activity	Molecular function
Cyclophilin	229	9,00E-23	AAG13968	249	Catalytic activity	Molecular function
	2275	1,00E-20	AAF69795	213	Catalytic activity	Molecular function
Exopolygalacturonase PGX1	pr0aaa12yk07scm1.1	1,00E-30	AAK81847	181	Catalytic activity	Molecular function
G protein alpha subunit	3233	4,00E-22	AAC49724	76	Binding	Molecular function
Glyoxaloxidase 1	1675	9,00E-15	CAD79488	352	Catalytic activity	Molecular function
G-protein beta subunit 1	402	9,00E-15	AAP55639	316	Binding	Molecular function
Guanine nucleotide exchange factor Cdc24	pr0aaa67yi20scm1.1	9,00E-19	AAO25556	283	Enzyme regulator activity	Molecular function
Guanyl nucleotide exchange factor Sql2	pr0aaa26ye11scm1.1	2E-14	AAO19638	319	Enzyme regulator activity	Molecular function
	pr0aaa94yf22scm1.1	6,00E-15	AAA02743	463	Binding	Molecular function
Imidazole glycerol phosphate dehydratase	2572	3,00E-17	AAB88888	121	Cellular process/metabolic process	Biological process
Isocitrate lyase	1103	2,00E-39	AAM89498	261	Catalytic activity	Molecular function
	pr0aaa36yl22scm1.1	1,00E-36	AAM89498	261	Catalytic activity	Molecular function
MAP Kinase	pr0aaa24yh13scm1.1	6,00E-29	AAO27796	342	Binding	Molecular function
	1219	4,00E-23	CAC47939	245	Binding	Molecular function
	908	2,00E-16	AAK49432	234	Binding	Molecular function
	955	8,00E-31	AAF15528	151	Binding	Molecular function
	374	5,00E-25	AAF15528	151	Binding	Molecular function
Mitochondrial glycoprotein, Mrb1	1454	9,00E-19	AAT81148	367	Multiorganism process	Biological process
NADH-ubiquinone oxidoreductase 49 kDa subunit, mitochondrial precursor	2040	3,00E-32	EAA69636	445	Multiorganism process/catalytic activity	Biological process/molecular function
Peroxisome biogenesis – Pex6 protein	2886	3,00E-31	AAK16738	226	Metabolic process/cellular process	Biological process
Pheromone receptor CPRa1p	660	2,00E-32	AAK31936	292	Signal transducer activity	Molecular function
Phosphatidylinositol 3-kinase	4039	1,00E-27	CAA70254	195	Catalytic activity	Molecular function
Polygalacturonase	3187	2E-11	CAA71246	247	Catalytic activity	Molecular function
Protein kinase	444	1,00E-16	AAW46354	360	Binding/calalytic activity	Molecular function
	2829	9,00E-48	AAB68613	85	Binding/calalytic activity	Molecular function
	2748	2,00E-23	AAC09291	158	Binding/calalytic activity	Molecular function
Protein mannosyltransferase	1910	1,00E-51	AAF16867	452	Catalytic activity	Molecular function
	pr0aaa54yd01scm1.1	8,00E-48	CAA67930	104	Catalytic activity	Molecular function
Putative branched-chain amino acid aminotransferase	1888	9,00E-21	AAD45321	157	Catalytic activity	Molecular function
Rab subfamily of small GTPases, Rsr1p	231	8,00E-26	CAC41973	339	Binding	Molecular function
	pr0aaa81ye23scm1.1	5,00E-22	CAC41973	339	Binding	Molecular function
	pr0aaa90yb05scm1.1	5E-10	CAC41973	339	Binding	Molecular function
	3235	1,00E-44	CAC41973	339	Binding	Molecular function
Ras-like small GTPases CaRho1	3583	4,00E-38	BAA24262	270	Binding	Molecular function
Topoisomerase I	1694	1,00E-17	AAB39507	80	Binding/catalytic activity	Molecular function
Transcriptional repressor	pr0aaa11yo22scm1.1	2,00E-20	AAB63195	211	Cellular process/metabolic process	Biological process
	pr0aaa104ym01scm1.1	9,00E-20	AAB63195	211	Cellular process/metabolic process	Biological process
Transmembrane protein	631	5,00E-42	AAD51594	267	Binding	Molecular function
	pr0aaa92yb19scm1.1	5,00E-32	AAD51594	267	Binding	Molecular function
	2916	7,00E-25	AAD51594	267	Binding	Molecular function
	pr0aaa47yd11scm1.1	7,00E-22	AAD51594	267	Binding	Molecular function
	1972	3,00E-19	AAD51594	267	Binding	Molecular function
	3153	1E-13	AAD51594	267	Binding	Molecular function
	pr0aaa62yh02scm1.1	7E-13	AAD51594	267	Binding	Molecular function
	2016	2E-11	AAD51594	267	Binding	Molecular function
Trehalose-6-phosphate phosphatase	2473	9,00E-49	AAN46744	322	Catalytic activity	Molecular function
	960	2,00E-21	AAN46744	322	Catalytic activity	Molecular function
Uac	pr0aaa75yc14scm1.1	9E-14	AAA57469	22	Catalytic activity	Molecular function
Urease	pr0aaa84yg09scm1.1	3E-13	AAC62257	194	Metabolic process	Biological process
	1663	4E-11	AAC62257	194	Metabolic process	Biological process
vacuolar (H+)-ATPase subunit	2474	2E-13	AAK81705	235	Localization	Cellular component
Virulence associated DEAD box protein 1	1516	3E-13	AAV41010	423	Binding	Molecular function
	2939	4E-12	AAV41010	423	Binding	Molecular function
	pr0aaa70ym18scm1.1	8E-11	AAV41010	423	Binding	Molecular function
Hypotethical protein	1112	1,00E-28	EAL03139	290	Binding	Molecular function
	1224	4,00E-16	EAL03139	290	Binding	Molecular function
	pr0aaa11yc08scm1.1	7E-13	EAL03139	290	Binding	Molecular function

### Sequences relevant to mating-types

Our cDNA library contained 70 sequences presenting a similarity (E-value ≤ 10^-10^) with genes belonging to, or linked to, the MAT loci in other fungi. According to the Gene Ontology classification, most of these sequences (61%) would have molecular functions. Thirteen sequences were similar to pheromone receptors, transporters or response factors, mainly from the other basidiomycete species *Coprinopsis cinerea, Schizophyllum commune, Ustilago maydis *and *Cryptococcus neoformans*. We also identified 332 sequences similar to genes regulating mating, morphogenesis and pathogenesis, such as the mitogen-activated protein kinase (MAPK) and the cAMP dependent protein kinase (PKA), components of the PKA/MAPK network in *U. maydis *[4]. Other sequences had a significant similarity to transcription factors, like the Prf1 of *U. maydis *[20], which are essential for the interconnection between the pathways of PKA and MAPK pathways.

The most interesting ESTs regarding the MAT locus of *M. violaceum *were those constituting the four unisequences similar to pheromone receptors. These four unisequences (the singlet pr0aaa87yh06 and the contigs 588, 2096 and 660) showed significant sequence similarity to each other but not enough to be assembled in a single contig. We designed primers (Table 2) within each of the four unisequences similar to pheromone receptors and performed PCRs on A1 and A2 sporidial lines of ten strains of *M. violaceum*. Amplification products were of higher size than expected from ESTs for 3 of the unisequences, indicating the presence of introns (Table 2). The amplifications corresponding to each of the four unisequences were specific of a single mating type (Table 2).

**Table 2:** Unisequences of *Microbotryum violaceum *blasting against pheromone receptors. For each of the four unisequences significantly blasting against pheromone receptors: best hits, primer designed for PCR amplification, expected size from the EST sequence, rough amplification size obtained, and mating type of the sporidia that gave amplification products.

	**Number of ESTs**	**Best hits**	**Primers**	**Contig size obtained from ESTs**	**Rough amplification size**	**Amplification in sporidia of mating type**
Contig588	3	Rcb1 of *Coprinopsis cinerea*	F^1^: GGAAGGCCATTACAAGAAAGG	350	500	A2 only
		Bbr2 of *Schizophyllum commune*	R^2^: TGTGCTTTTCGCTCTTAGCA			
Contig660	4	Rcb2 of *C. cinerea*	F: ACGATTCCAGTAGGCGTGAA	551	800	A1 only
		B alpha 8 of *S. commune*	R: CTGCGTCACGATACCTTTCTT			
Contig2096	3	Bbr2 of *S. commune*	F: TCCTTTGTCACGACAAGCAC	213	220	A1 only
		Rcb3 of *C. cinerea*	R: CCAATTTTCACGCCTACTGG			
Singlet pr0aaa87yh06	1	Rcb1 of *C. cinerea*	F: ATCAGAATATGACGGCAGCA	383	600	A2 only
		Bbr2 of *S. commune*	R: AAGAAAGGGAACTCCAAATGC			

^1 ^F: Forward primer

^2 ^R: Reverse primer

Furthermore, the p-distance [21] showed that sequences of singlet pr0aaa87yh06 and contig 588 were highly similar (p = 0.273) and identical on the second halves of the sequences (p = 0.000). Inspection of the chromatograms showed that one of the 3 ESTs assembled in the contig 588 was of very poor quality on the first half of the sequences, suggesting that the singlet pr0aaa87yh06 and contig 588 were actually probably transcripts of the same gene. This was checked by designing primers on the most different parts of the two unisequences, which amplification products indeed yielded identical sequences, including the intronic parts.

These two sequences were less similar to the contigs 2096 and 660 (p = 0.702 and 0.793 respectively). The contigs 2096 and 660 overlapped only on 25 bp, but aligned one to each other perfectly at their edges (p = 0.000), suggesting that they represent ESTs from the same gene. Contigs 2096 and 660 were not assembled into a single contig because the region of overlap with sufficient PHRED quality sequence was too short. The PCR performed using the forward primer of the Contig 2096 and the reverse primer of the Contig 660 (Table 2) yielded a single amplification product whose sequence read without apparent heterogeneity on the chromatograms. This indicates that the contigs 2096 and 660 indeed correspond to the same pheromone receptor.

**Figure 3:**
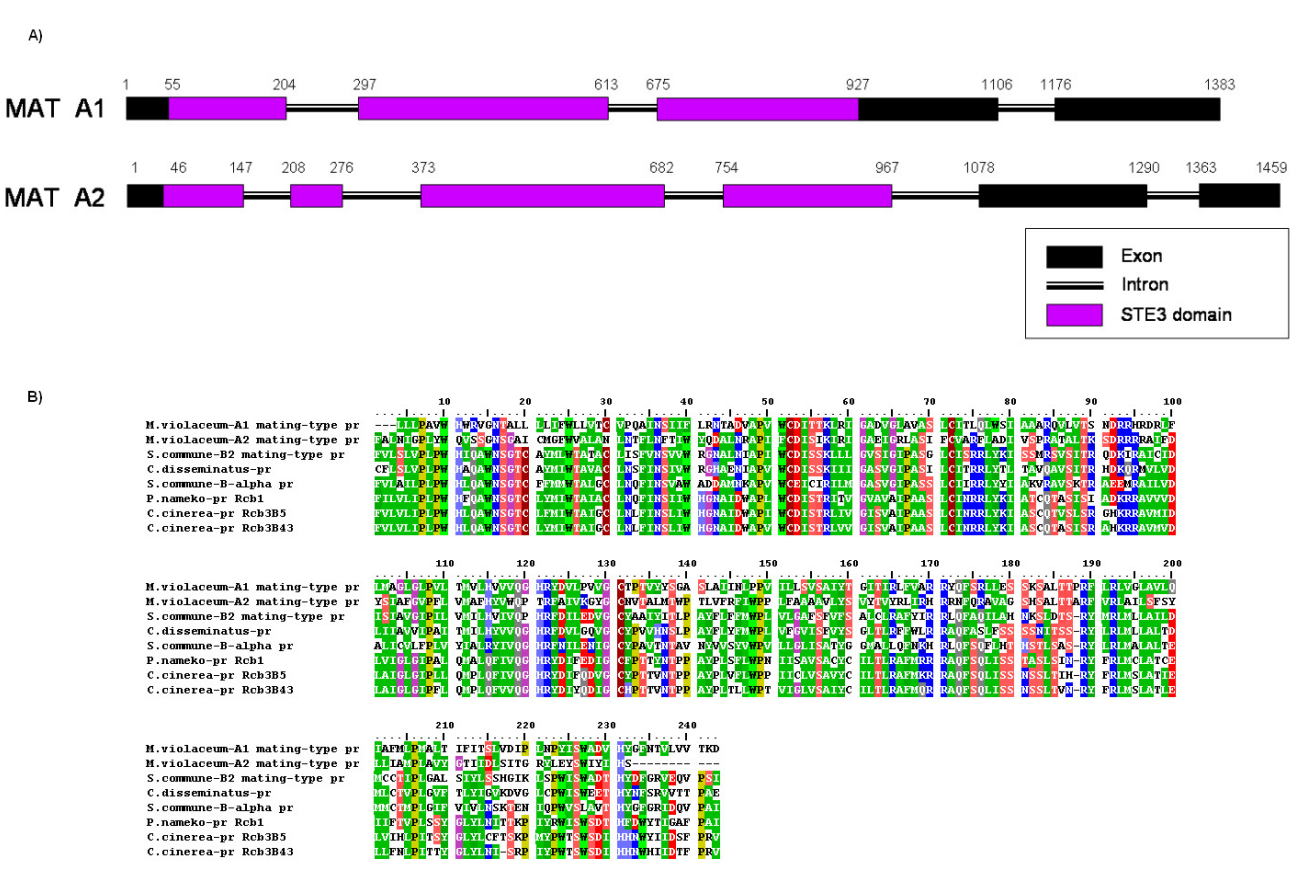
**Putative pheromone receptors in *Microbotryum. violaceum***. A) Diagram of the two putative pheromone receptor genes identified in the EST library of *Microbotryum violaceum*, respectively linked to the A1 and A2 mating type. B) Alignment of the two putative pheromone receptors of *Microbotryum violaceum *with the most similar published protein sequences of other fungi: B2 and B-alpha of *Schizophyllum commune*, the transmembrane pheromone receptor of *Coprinellus disseminatus *and Rcb3B5 of *Coprinopsis cinerea*.

*Microbotryum violaceum *thus appears to carry a single pheromone receptor at the A1 locus and a single pheromone receptor at the A2 locus, which would be in agreement with its bipolar status. In contrast, tetrapolar species such as *C. cinereus *and *S. commune *have several pheromone receptors at each of the alternate forms of the B mating type locus [22,23]. A genome walking approach allowed us to obtain the complete sequence of the putative A1 and A2 pheromone receptors of *Microbotryum violaceum *(Figure 3A; Genbank accession numbers EF584742 and EF584741, respectively for the A1 and A2 pheromone receptors).

The putative pheromone receptors identified in our cDNA library did not show highly significant similarity to the pheromone receptors of *U. maydis *and *U. hordei*, which explains why they hybridized only weakly on Southern blots [24], and why cloning attempts of the *M. violaceum *mating type locus by designing degenerate primers from the *U. maydis *sequences have failed (T. Giraud, unpublished).

The cloning of the complete mating type locus of *M. violaceum *is currently under way, starting from the pheromone receptors obtained in the present library. The complete sequence of the mating-type locus will allow identifying the organisation and composition of this genomic region, and thus understand how the transition occurred between tetrapolarity and bipolarity in *M. violaceum *or its ancestral lineages. One tentative hypothesis given the data at hand is that it exists a single allele of each mating type locus and that the two mating type loci are linked, as in *U. hordei *[7]. Recombination is indeed suppressed along most of the sex chromosomes in *M. violaceum *[25].

### Other sequences relevant to pathogenesis

A total of 70 sequences had a high similarity to genes shown experimentally to play a role in pathogenicity in other fungi (Table 2). An important class of proteins in pathogenicity is the secretome, which play important roles in penetration and colonization of plant tissues [26]. No sequence in MICROBASE presented high similarity with genes encoding cell wall-degrading enzymes, such as lyases, lipases, proteases, and we detected only two polygalacturonases. Plant pathogens that kill host cells, like *Magnaporthe grisea *and *Fusarium graminearum*, contain in their genome many genes involved in degradation of cell tissue. In contrast, it is not surprising to find a reduced number of genes involved in such hydrolytic functions in fungi with a biotrophic life style in which host damage is minimized, like *M. violaceum*. Similar conclusions have been drawn from the complete genome sequence of *Ustilago maydis *[27], which also has a biotrophic life style. The genome of *U. maydis *contained in contrast numerous secreted proteins with unknown functions, and even with no similarity to any other proteins in the databases. The total number (594) of proteins predicted to be secreted in MICROBASE was similar to that in the genome of *U. maydis *[27], and the percentage of secreted proteins without a significant hit in databases was also very high (86.4% in MICROBASE). This suggests that the specific and intimate relationships between biotrophic fungi and their host plant select for specific secreted functions.

In several fungi, the cAMP signalling and two MAP kinase pathways have been implicated in regulating various plant infection processes, in particular in monocot-infecting smuts [28]. Several contigs of *M. violaceum *were similar to enzymes of these molecular pathways, including G proteins, protein kinases and Ras proteins. In *U. maydis *for instance, disruption of *Ras2 *resulted in loss of pathogenicity and dramatic changes in cell morphology [29]. Another important molecular pathway in pathogenic fungi is the Calcineurin/cyclophilin signalling [30], for which we also detected putative genes in the MICROBASE. Other important molecules involved in pathogeniticy belong to the secondary metabolism which includes P450 genes, such as the putative ones present in the MICROBASE, or the small peptides synthetized by nonribosomal peptide synthases (NRPS). We detected contigs similar to NRPS, such as the one similar to CPS1 [31].

### Expressed transposable elements

**Figure 4:**
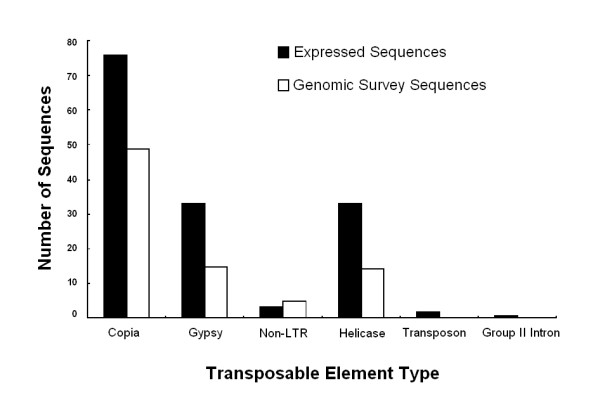
**Comparison of expressed and genomic copies of *Microbotryum violaceum *transposable elements**. Class I elements are represented by the Copia, Gypsy, and Non-LTR (long-terminal repeat) categories; Class II elements are represented by the Helicase and DNA Transposon categories. The Group II Intron category corresponds to a mitochondrial mobile element. The data on genomic survey sequences are from ref [12].

Our library presented 148 unisequences with significant similarity to transposable elements (TE), with an additional 10 showing putative or weak similarity to TEs. The 148 unisequences, when categorized by the major types of Class I (RNA-based replication) and Class II (DNA-based replication) transposable elements, were in similar relative frequencies as TEs from the *M. violaceum *genomic survey [12] (Figure 4). Putative Class II DNA transposons and a maturase sequence from a mitochondrial Group II Intron were also identified among the expressed sequences, but were not found in the prior genomic survey. Although Hood et al. [32] showed that the RIP (repeat-induced point mutation) genome defense has been very active in *M. violaceum*, our results suggest that the transposable elements can escape this genomic mechanism of defense to some extent, at least regarding the transcriptional activity. In fact, there is evidence of RIP mutation among the expressed TE sequences; five unisequences could be aligned with the genomic consensus of copia-like integrase gene from a prior analysis of RIP in *M. violaceum *[32], and among these alignments mutations at RIP recognition sites were 2 to 3 times more frequent that to any other sites.

Prior studies have reported that some unidentified transposable elements may be active only during mitosis, whereas others would be active during meiosis [33], and the conditions under which our library was built may therefore lead to an underestimation of the TE transcriptional activity. A more specific study is required to understand the importance of the RIP mechanism in the accumulation of transposable elements in the genome of *M. violaceum*, especially as RIP-affected and non-functional TE copies may still be transcribed. The comparison of TE transcripts in the MICROBASE with the genomic copies should be interesting to estimate the impact of the RIP defense mechanism in *M. violaceum*. We did not identify any EST similar to the RID (RIP defective) DNA methyltransferase gene required for RIP in *Neurospora crassa *[34], although we detected several sequences similar to methyltransferases.

## Conclusion

This study, providing the first extensive genomic dataset on *M. violaceum*, has permitted the detection of many genes putatively involved in mating, some of which were shown to be linked to the mating-type locus, and also many genes possibly involved in pathogenesis. Studies of reverse genetics are however required to validate these putative biological functions. Studies of comparative genomics among fungi should also benefit from the existence of resources such as the MICROBASE [35]. This extensive database will not only allow comparing the sequence evolution among species, but also searches for the presence of genes and the numbers for gene families. Such comparative approaches yield valuable insights into the evolution of host-pathogen interactions [35]. Furthermore, it is now possible to clone and sequence the whole mating type locus of *M. violaceum*, allowing elucidating its organization. Comparison with the mating type loci of other basidiomycetes will then provide insights into its evolution, in particular into the mechanism of the transition between tetra- and bipolarity. Finally, the high expression level of transposable elements raises questions about the importance of the RIP genome defense, and how it can be escaped.

## Methods

### *Microbotryum violaceum *strain and culture conditions

Teliospores from the strain 100.02 of *M. violaceum*, collected from the host *Silene latifolia *in 2001 in the Alps, near Tirano in Italy, was plated on GMB1 medium [36]. On such nutritive media, diploid teliospores germinate and produce haploid sporidia of the two mating type A1 and A2. A1 and A2 sporidia lines from the strain 100.02 were identified by pairing with existing stocks of known mating type.

A mixed suspension of A1 and A2 sporidia (250 μL of each) was plated on water agar supplemented with α-tocopherol (10 IU/g) and incubated at 4°C for one week. These conditions of low nutrients with α-tocopherol are thought to mimic the host plant surface for the fungus, because sporidia conjugate and produce hyphae of a few cells [37]. This was checked using a light microscope (400×).

### RNA isolation, cDNA library construction and sequencing

Total RNA was extracted from conjugated cells and hyphae using the Trizol reagent following the manufacture protocol (Invitrogen, The Netherlands). Extractions yielded 50 μg–500 μg of total RNA. Poly (A^+^) RNA was purified using the mRNA Absolutely Purification Kit (Stratagene, CA). The SuperSmart cDNA Synthesis Kit (Clontech, CA) was used to synthesize cDNA, and the library was normalized using the Trimmer kit (Evrogen, Moscow) that reduces the quantity of the most abundant cDNA copies. cDNAs were ligated into the pGEM-T vector (Promega, WI). To test the quality of the ligation, we transformed ultracompetent cells (XL10-Gold, Stratagene, CA) and amplified inserts from 100 clones. The average size of inserts was 500 bp. Individual colonies were examined using the blue-white selection for the vector giving >50% of vector with inserts and an estimate of 2.0 × 10^5 ^cfu. Forty thousand clones were then sequenced in one direction by the Genoscope (Evry-France) using the primer of cDNA synthesis kit (SMART II A Oligonucleotide 5'-AAG CAG TGG TAT CAA CGC AGA GTA CGC GGG-3').

### Sequence analyses and EST clustering

Raw sequence data were cleaned from vector and adaptor sequences. Contaminating plasmid sequences, such as *E. coli*, mitochondrial or ribosomal fungal sequences were removed from the analyses. PHRED software [38,39] was used for base-calling the chromatogram trace files. Only sequences with a PHRED score over 20 on at least 100 bp were released in the EST division of the EMBL-EBI (European Molecular Biology Laboratory – European Bioinformatic Institute) Nucleotide Sequence Database.

ESTs were aligned and assembled into contigs using the CAP3 software [40] when the criterion of a minimum identity of 95% over 50 bp was met. When an EST could not be assembled with others in a contig, it remained as a "singlet". The contigs and the singlets should thus correspond to sequences of unique genes, and will be called hereafter "unisequences". The consensus sequences of the contigs and the sequences of the singlets were compared to the sequences in the GenBank database and in the Uniprot database using the tBLASTx and the BLASTx algorithms [41]. Unisequences showing significant similarity (E-value <= 10^-4^) to database entries were annotated using the most significant matches. Unisequences were also classified into Gene Ontology functional categories [42] based on BLAST similarities to known genes of the NCBI nr (non-redundant) protein database and using the Blast2GO annotation tool [43]. Sequences were also compared to the pathogenicity genes assembled in the PHI database [2,44] and to the genes linked to the mating-type in other fungi using a manually built list of such genes. The sequences showing significant similarity to transposable elements were also recorded. WoLF PSORT version 2.0 [45] was used to predict protein localization using the higher prediction score for external compartments. Finally, using a modified version of the ESTIMA tool [46] we developed a public database named MICROBASE, dedicated to *Microbotryum violaceum *EST management and analysis. This database includes information on EST sequences, contigs, annotations, gene ontology functional categories and search programs to compare similarities of any sequence against the database. MICROBASE is accessible freely through a web interface [18].

### Amplification of putative pheromone receptors

Primers were designed in the four unisequences with significant sequence similarity to pheromone receptors (Table 2) and amplifications were performed on DNA extracted from sporidia of known mating type, from ten different strains of *M. violaceum*, of various geographical origins. DNA was extracted from single-sporidial colonies using the Chelex (Biorad) protocol [47]. PCR amplifications were performed using a PTC 100 thermal cycler (MJ Research), with 65°C as the annealing temperature, for the amplification to be as specific as possible, using the Qbiogene (Irvine, CA) *Taq *polymerase following the manufacturer recommendations.

### Genome walking

High quality genomic DNA was isolated from a *Microbotryum violaceum *strain from *S. latifolia*. The DNA was digested by blunt end cutting enzymes (*Dra*I, *Pvu*II, *Eco*RV and *Stu*I) provided in the Universal GenomeWalker kit (BD Biosciences, Clontech, USA). The digested DNA was then purified and ligated overnight with the adaptors provided in the kit. The genome walking approach was followed according to the manufacturer instructions.

## Authors' contributions

TG and RY contributed to the conception and design of the study, to the acquisition and analysis of data, to coordination of the study, and were involved in drafting the manuscript. SM, HC, MEH participated in data analysis and drafting of the manuscript. FR and AG were involved in data analysis. CR and PW carried out the sequencing and first data analysis. BD performed sequences and analyses of the pheromone receptors. All authors read and approved the final manuscript.
